# Targeting DNA Damage Response in Prostate and Breast Cancer

**DOI:** 10.3390/ijms21218273

**Published:** 2020-11-04

**Authors:** Antje M. Wengner, Arne Scholz, Bernard Haendler

**Affiliations:** Preclinical Research, Research & Development, Pharmaceuticals, Bayer AG, Müllerstr. 178, 13353 Berlin, Germany; antje.wengner@bayer.com (A.M.W.); arne.scholz@bayer.com (A.S.)

**Keywords:** DNA repair, DNA damage response, hormone-dependent, prostate cancer, breast cancer, radiation, PARP-1, ATR, ATM, DNA-PKcs

## Abstract

Steroid hormone signaling induces vast gene expression programs which necessitate the local formation of transcription factories at regulatory regions and large-scale alterations of the genome architecture to allow communication among distantly related cis-acting regions. This involves major stress at the genomic DNA level. Transcriptionally active regions are generally instable and prone to breakage due to the torsional stress and local depletion of nucleosomes that make DNA more accessible to damaging agents. A dedicated DNA damage response (DDR) is therefore essential to maintain genome integrity at these exposed regions. The DDR is a complex network involving DNA damage sensor proteins, such as the poly(ADP-ribose) polymerase 1 (PARP-1), the DNA-dependent protein kinase catalytic subunit (DNA-PKcs), the ataxia–telangiectasia-mutated (ATM) kinase and the ATM and Rad3-related (ATR) kinase, as central regulators. The tight interplay between the DDR and steroid hormone receptors has been unraveled recently. Several DNA repair factors interact with the androgen and estrogen receptors and support their transcriptional functions. Conversely, both receptors directly control the expression of agents involved in the DDR. Impaired DDR is also exploited by tumors to acquire advantageous mutations. Cancer cells often harbor germline or somatic alterations in DDR genes, and their association with disease outcome and treatment response led to intensive efforts towards identifying selective inhibitors targeting the major players in this process. The PARP-1 inhibitors are now approved for ovarian, breast, and prostate cancer with specific genomic alterations. Additional DDR-targeting agents are being evaluated in clinical studies either as single agents or in combination with treatments eliciting DNA damage (e.g., radiation therapy, including targeted radiotherapy, and chemotherapy) or addressing targets involved in maintenance of genome integrity. Recent preclinical and clinical findings made in addressing DNA repair dysfunction in hormone-dependent and -independent prostate and breast tumors are presented. Importantly, the combination of anti-hormonal therapy with DDR inhibition or with radiation has the potential to enhance efficacy but still needs further investigation.

## 1. Introduction

Genomic stability is essential for all living organisms and is safeguarded by different complex and coordinated DNA damage response (DDR) pathways. These mechanisms protect cells against intrinsic insults such as reactive oxygen and nitrogen species or DNA replication errors as well as against extrinsic insults, mainly ultraviolet light and ionizing radiation causing single-strand breaks (SSBs) or the more severe double-strand breaks (DSBs) in the DNA [1,2,3]. Another essential role of the DDR is the repair of damage originating from stress during DNA replication and gene transcription [4,5,6,7]. Steady progress has been made in understanding the multistage response to DNA damage, which includes detection by sensor proteins, control of cell cycle progression, recruitment and activation of effector proteins, and finally repair of the damage [3,8,9,10,11]. A role of microRNAs in this process has additionally been recognized [12]. For instance, miR-34 family members are upregulated following DNA damage and regulate the expression of checkpoint genes. Also, upregulation of miR-146 which reduces BRCA1 expression has been reported. The DDR machinery is intimately linked to cellular senescence and also regulates apoptotic pathways which will exit cells permanently from the cell cycle or eliminate them by programmed cell death in case the DNA lesion cannot be repaired and genome integrity is not safeguarded [10,13].

Cancer cells are characterized by genomic instability which favors the accrual of driver mutations and the expansion of tumor heterogeneity [14]. This feature has been addressed for many years by cytotoxic chemotherapy and radiation treatment which cause severe DNA damage in fast-dividing cancer cells. Tumors frequently harbor alterations in DDR pathways leading to genomic instability that can promote tumorigenesis and cancer cell growth, as reflected in the acquisition of driver mutations [9,10,15,16,17]. Concurrently, defects in DDR signaling, such as alterations in essential DDR genes [18,19] or changes in DDR gene expression, for instance, mediated by epigenetic silencing mechanisms [20,21], may increase the dependence on other DDR actors for survival. The steadily increasing knowledge about the mechanisms involved in these processes allowed the identification of potential weaknesses in tumors that can be addressed with innovative targeted therapies following the concept of synthetic lethality in which two pathway defects, that alone are non-toxic, become lethal when combined [8,10,18,22].

Prostate cancer is originally dependent on androgen when diagnosed, and mainstay medications used are androgen-deprivation therapy, androgen receptor (AR) antagonists, and androgen synthesis inhibitors [23,24,25]. Unfortunately, resistance often follows, mainly due to the amplification of the AR gene and overexpression, AR mutations and splice variants, and increased androgen synthesis [26,27]. Additional resistance mechanisms involving for instance the PI3K pathway have been reported [28]. Concerning breast cancer, approximately two-thirds of patients express estrogen receptor (ER) α and are treated with ERα antagonists or aromatase inhibitors [29,30]. Treatment resistance linked to the emergence of ERα-negative tumor cells may occur at some timepoint, necessitating the switch to other therapies [29]. Prostate and breast tumors often have mutations affecting the DDR, both in germinal and somatic tissues. Concerning the prostate, single-nucleotide polymorphisms (SNPs) in different DDR genes have been linked with increased cancer risk. Germline mutations leading to inactivation of DDR genes are found in up to 20% of primary tumors and are correlated to early onset [31,32,33,34,35]. A survey of 131 primary and 37 metastatic prostate tumors detected changes in many DDR genes with, however. A large variability among samples [36]. Mutations in genes encoding ataxia–telangiectasia-mutated (ATM) kinase, BRCA2, and poly(ADP-ribose) polymerase 1 (PARP-1) represent the most frequent alterations and are associated with aggressive disease and worse outcome. Additional mutations were reported in RAD51 and additional DDR genes [37]. Mutations in DDR factors generally increase during tumor progression and are found in 35–40% of metastatic castration-resistant prostate cancer (mCRPC) [32]. Here, also, ATM and BRCA2 are among the most frequently altered genes. Another study showed that expression of DNA-dependent protein kinase catalytic subunit (DNA-PKcs) was reduced in 51% of prostate cancer biopsies [38]. Interestingly, defects in mismatch repair are associated with increased T-cell infiltration and immune transcripts in a subgroup of prostate tumors [39]. Cyclin-dependent kinase 12 (CDK12) plays an essential role in transcription regulation and mRNA splicing, and is mutated in 1–2% of localized prostate cancer and 4–7% of mCRPC [40,41]. It controls proper expression of several genes involved in homologous recombination (HR) such as BRCA1, BRCA2, and ATM and represents an important link between transcription regulation and genome stability [42,43]. CDK12 biallelic inactivating mutations define a distinct subtype in advanced mCRPC. Loss of CDK12 is associated with genomic instability and focal tandem duplications, leading to increased gene fusions and marked differential gene expression, especially in genes involved in cell cycle and DNA replication [40]. Tandem duplications have also been described for an enhancer of the AR potentially responsible for disease progression on androgen pathway inhibitors [44,45].

Alterations in DDR genes have also been reported in breast cancer. Approximately 10% of cases are linked to germline defects in BRCA1 or BRCA2, whereas mutations in other DDR genes, such as those encoding CHK2 and RAD51, are rarer [46,47]. Another study detected germline or somatic BRCA1 or BRCA2 inactivating mutations or BRCA1 promoter methylation in 90 out of 560 breast cancer samples [48]. An observational study in human epidermal growth factor receptor 2 (HER2)-negative breast cancer patients showed that germline BRCA mutations were associated with earlier age of diagnosis and family history [49]. Importantly, not all variants of BRCA1 or BRCA2 have been linked to pathogenicity, thus gene expression signatures to address this have been proposed [50]. Changes in other DDR genes have additionally been found. Expression of DNA-PKcs was reduced in 57% of early breast cancer cases [51]. In a subgroup of metastatic breast cancer patients expressing the ERα and progesterone receptor (PR) but not HER2, somatic mutations in BRCA1, BRCA2 or ATM were found in 4% of cases [52]. The CDK12 gene was amplified or mutated in over 10% of invasive breast cancer samples [53]. Concerning triple-negative breast cancer (TNBC), a detailed survey where 56 DDR genes were analyzed revealed a heterogeneous pattern, including mutations in BRCA, non-BRCA HR, and non-HR genes [54]. The analysis of four TNBC cell lines showed overexpression of several proteins involved in DNA repair including PARP-1 [55]. Another study indicated that several DDR genes were regulated by CDK12 in a TNBC cell model [56].

All these findings led to extensive investigations to evaluate the potential of DDR-targeting drugs for prostate and breast tumors which culminated in the approval of PARP-1 inhibitors for breast cancer and, very recently, also for prostate cancer [57,58,59]. Compounds addressing several other essential actors of the DDR are currently being evaluated in clinical studies both in hormone-dependent and -independent stages of tumor growth, and are discussed below.

## 2. DDR and Steroid Hormone Receptor Pathways

### 2.1. General Aspects

Different repair mechanism pathways are activated, depending on the type of DNA damage. A SSB is more frequently observed but comparatively less damaging to cells. It is resolved by base excision repair (BER), mismatch repair (MMR) or nucleotide excision repair (NER) [10,60,61]. Intra- and inter-strand crosslinks introduced by DNA-damaging factors are resolved by the Fanconi anemia (FA) pathway [62]. DSBs are the most deleterious DNA lesions for living cells, especially when they occur in clusters [63]. These lesions are repaired by two main mechanisms, namely, HR, where the original DNA is resynthesized in a seamless way based on the sister chromatid template [10,61,64], and non-homologous end-joining (NHEJ), where severed DNA ends are ligated together again, but deletions are also introduced [65,66]. HR and NHEJ have essential but also overlapping roles in the maintenance of chromosomal integrity during the cell cycle in vertebrate cells [66].

### 2.2. Transcription-Coupled DNA Repair

Regions where intense transcription takes place have a loosened chromatin structure and undergo large-scale changes which make them more liable to DNA damage. Transcription-blocking DNA lesions are sensed by the RNA polymerase II complex and lead to stalling, which is followed by cell cycle arrest and apoptosis if it persists [4,6,7,67,68]. These processes are furthermore controlled by epigenetic mechanisms, such as histone acetylation and methylation marks, which affect the recruitment and stability of local protein complexes dedicated to DNA repair [69]. Prostate and breast tissues are particularly liable to this stress due to the sustained transcriptional activity elicited by steroid hormones [4,67]. Persistent signaling by the AR or ER requires numerous transcription factors and cofactors to interact with gene regulatory regions, leading to topoisomerase II-induced DSBs which are efficiently repaired by the NHEJ pathway [70,71]. These breaks are recognized by Ku70 and Ku80, leading to recruitment of PARP-1, DNA-PKcs, and ATM for repair.

Distant regulatory regions, such as enhancers and super-enhancers. are essential for full, sustained transcriptional activity. Super-enhancers are a newly described class of large-size cis-regulatory elements responsible for cell-type identity which, however, can be converted into oncogenic players [72,73,74]. They are highly loaded with the mediator complex and the bromodomain protein BRD4, and they form long-range interactions by chromosomal looping and assemble into local transcription factories [73,75,76]. Super-enhancers are inside stable cellular compartments and form phase-separated liquid condensates with unique properties [76,77]. SNPs associated with prostate or breast cancer are enriched at super-enhancer regions bound by BRD4 and marked by H3K27 acetylation [78]. Active gene regulatory regions often undergo DNA breaks, especially when they have turned into oncogenic drivers as recently evidenced [79]. In steroid-dependent cells, these regulatory elements are highly bound by ligand-activated AR or ERα for full transcriptional activity [80,81]. AR-loaded enhancers bound by DNA topoisomerase I for local DNA nicking and by components of the DDR pathway have been identified [82]. In addition, there is recruitment of the ATM and Rad3-related (ATR) kinase, the MRE11A/RAD50/NBS1 (MRN) complex and of other players of the DDR process to ensure that DNA breaks are properly resolved [82]. Tissue-specific super-enhancers with potentially increased sensitivity to DNA DSBs were identified as rearrangement hotspots in breast cancer samples [83]. High occurrence of DSBs due to the presence of DNA topoisomerase I activity was observed at super-enhancers of a breast cancer cell line. These sites are highly loaded with transcriptional enhancer factor domain (TEAD) transcription factors which interact with the DNA repair protein RAD51 to support local mending [84].

### 2.3. Cross-Talk between the AR and the DDR Pathways

Accumulating data show that the AR pathway regulates DNA repair factors. A total of 32 genes associated with DNA repair are bound by androgen-stimulated AR as demonstrated in the LNCaP prostate cancer cell line model [85]. Both androgen deprivation and AR antagonist treatment lead to inhibition of DNA damage pathways. In another transcriptomics analysis of different prostate cancer cell lines, a subset of DDR genes was also found to be regulated by androgen [36]. Androgen-dependent recruitment of cyclin D1 and formation of a complex with RAD51 giving rise to DDR was shown in another study [86]. Also, a positive regulation of DNA-PKcs, as well as of XRCC4 and XRCC5, with essential functions in NHEJ, was reported [87]. Another study demonstrated that androgens regulate the expression of NKX3.1, a tumor suppressor that activates ATM to recruit DNA repair actors involved in HR [88]. In line with these observations, AR antagonists impair the DDR at several levels. Blockade of AR function leads to reduced expression of several HR-associated genes such as BRCA1, RAD54L and RMI2, so that sequential treatment of a prostate cancer xenograft with the AR antagonist, enzalutamide, and the PARP-1 inhibitor, olaparib, strongly suppresses tumor growth [89]. Androgen deprivation results in elevated activity of the TLK1B/NEK1/ATR/CHK1 pathway in prostate cancer cells [90] and enzalutamide treatment reduces CDC6/ATR/CHK1 signaling [91]. The AR antagonist apalutamide reduces NHEJ-dependent recombination [85]. Also, decreased Ku70 levels were found in prostate tissues from men having undergone androgen-deprivation therapy (ADT) [92].

Conversely, DNA repair proteins also modulate AR function. DNA-PKcs is found at regulatory elements bound by the AR and stimulates its activity, thus eliciting changes in the transcriptional program and enhancing tumor progression [87]. On the other hand, AR activity is reduced following blockade of DNA-PKcs expression or activity. A recent study further elaborated on the cross-talk between DNA-PKcs and the AR, and highlighted the potential of combining respective inhibitors in patient-derived prostate cancer explants [93]. PARP-1 is also needed for full transcriptional activity of the AR and favors interaction at target genes [94]. The mediator of DNA damage checkpoint protein 1 (MDC1) facilitates the interaction between the AR and the histone acetyltransferase GCN5 which increases local histone acetylation and gene activation, thus promoting cell proliferation [95].

### 2.4. Cross-Talk between the ERα and the DDR Pathways

Estrogens stimulate several DDR pathways protecting against DSBs, as demonstrated in breast cancer models [96]. Estrogen-mediated DDR involving cyclin D1 and RAD51 has been reported [86]. ERα activates DNA-PKcs expression, thus increasing the ability of breast cancer cells to repair DSBs [97]. ERα stimulates NBS1 expression which is involved in HR and NHEJ, and protects cells from radiation-induced damage [98].

DNA repair proteins also control ERα function. PARP-1 directly binds and adds poly(ADP-ribosyl) groups to the ERα, which is necessary for interaction with target regulatory regions and full gene expression [99]. Following estrogen activation, DNA-PKcs forms a complex with ERα leading to phosphorylation and stabilization [97,100]. MDC1 binds to ERα and is recruited at target genes, thus increasing the transcriptional activity and ultimately breast cancer cell proliferation [101].

## 3. Targeting the DDR for Treatment of Prostate and Breast Cancer

### 3.1. PARP-1 Inhibitors

PARP-1 is the main member of the PARP family of nuclear proteins. It detects SSBs, induces a post-translational poly (ADP-ribosyl)ation (PARylation) to modulate chromatin structure, and guides the repair pathway by recruiting numerous factors to the damaged site [102,103]. It is also involved in DNA replication by controlling the elongation process and detecting disrupted forks [104]. The high frequency of tumors with BRCA1 or BRCA2 deficiency and their dependency on PARP-1 function has prompted intensive research efforts towards the identification of specific inhibitors. Potent and selective PARP-1 inhibitors, mostly acting as NAD+ competitors and blocking PARylation as well as inducing PARP trapping at the DNA, have been described and used to validate the underlying rationale [103,105]. Several PARP-1 inhibitors are now approved as monotherapy and additional clinical trials, including combination studies, are ongoing (Table 1). Olaparib was the first PARP-1 inhibitor approved, initially for advanced ovarian cancer with mutated BRCA, and then later also for breast cancer [106]. Very recently, olaparib and rucaparib have also received approval for mCRPC patients with HR repair or BRCA mutations, respectively, based on successful clinical trials [106,107,108]. Numerous clinical studies evaluating olaparib or other PARP-1 inhibitors as a single agent or combined with drugs, such as abiraterone, radium-223 or immune checkpoint inhibitors, are currently ongoing in mCRPC cohorts [109,110]. Serious adverse events have, however, been reported in patients receiving a combination treatment with olaparib and abiraterone [111]. The PARP-1 inhibitors rucaparib and talazoparib are also approved for BRCA-mutated breast cancer, and more clinical studies are currently ongoing in this indication [105,112]. Niraparib and veliparib are presently in late-stage clinical trials for prostate and breast cancer [105]. Clearly, PARP-1 inhibitors represent a novel treatment option for cancer patients with certain DDR alterations; however, upfront and acquired resistance, mainly due to the restoration of HR repair, are frequently observed after treatment [113]. Described resistance mechanisms include restoration of HR repair, protection of stalled replication forks caused by BRCA1/2 inactivation, and downregulation of 53BP1 gene expression [113,114].

### 3.2. DNA-PKcs Inhibitors

The DNA-PKcs is recruited and activated by the Ku70/80 heterodimer bound to DSBs and promotes NHEJ [8]. In this process, direct ligation of DNA breaks without the requirement of a sister chromatid or homologous chromosome is performed. A diverse collection of damaged ends is thereby repaired with efficient kinetics, but this mechanism has the disadvantage of being error prone. Increased DNA-PKcs expression is observed in a large fraction of late-stage tumors, including prostate and breast cancer [51,87], and is associated with poor outcome and resistance to radiation treatment or chemotherapy [115]. Several reversible and irreversible DNA-PKcs inhibitors with different chemical structures have been described [115]. NU7441 has strong anti-proliferative activity in different prostate cancer models, also in the absence of exogenous DNA damage, and exhibits synergy with enzalutamide [93]. The compound increases sensitivity to radiation of different tumor types, including breast cancer models [116]. Several selective DNA-PKcs inhibitors have reached the clinic (Table 2). Nedisertib (M3814) is currently in phase 2 for different indications, including mCRPC where a combination with radium-223 is evaluated. AZD7648 is a selective and potent DNA-PKcs inhibitor which increases the anti-tumor effects of chemotherapy and radiation [117,118]. It is currently tested in monotherapy and in combination with olaparib or with doxorubicin in patients with advanced cancer in a phase 1/2 study. VX-984 was already evaluated in a dose–escalation study in solid tumor subjects several years ago but no ongoing clinical study has currently been reported [115]. CC-115 is a dual DNA-PKcs and mTOR inhibitor displaying additive-to-synergistic efficacy with enzalutamide in different cell lines and explants derived from prostate cancer [93]. It is presently evaluated in combination with enzalutamide in a phase 1b study in CRPC patients [119].

### 3.3. ATR Inhibitors

ATR plays a central role in DNA repair, senses stressed replication forks, and orchestrates a multifaceted response to DNA replication stress [8,120]. This response is essential to ensure completion of DNA replication and maintenance of the integrity of the genome as indicated by the embryonic lethality observed in mice upon ATR depletion [121]. ATR is activated by various genotoxic stresses including stalled replication forks at single-strand DNA regions coated by replication protein A (RPA) or RAD17 [10]. This elicits activation of CHK1, degradation of CDC25A, and cell cycle arrest to allow DNA repair. Besides replication stress response, ATR also operates during HR-mediated DSB repair as well as at inter-strand and cross-link repair [122,123]. As ATR is essential for cell survival, it represents a valuable target for cancer treatment, especially in the context of ATM mutations [124]. Several ATR inhibitors are currently in early clinical testing (Table 2). Berzosertib (M6620) was initially profiled in lung xenograft models, where it showed strong efficacy when combined with cisplatin [125]. It was evaluated in solid tumor subjects following intravenous infusion as a single agent or combined with the DNA-damaging agent carboplatin, and an objective response was observed for one patient in each group [126]. Several phase 2 studies are currently ongoing with one focusing on mCRPC and one on breast cancer [125]. Ceralasertib (AZD6738) has preclinical anti-tumor activity mainly in combination therapy in several tumor types of different indications, especially in those with p53 or ATM deficiencies [120,127,128]. Clinical studies show that ceralasertib combined with carboplatin, olaparib or durvalumab leads to objective responses in all groups; however, no single-agent efficacy was reported [10]. The compound is currently being evaluated in several phase 1 or 2 trials in prostate and breast cancer including combination studies with olaparib in mCRPC patients [125]. M4344 shows strong anti-tumor efficacy in preclinical tumor models as a single agent or combined with talazoparib [129]. Two phase 1 clinical trials have recently been initiated for evaluation of this compound in solid tumors including ovarian cancer [125]. BAY 1895344 is a highly potent and selective oral ATR inhibitor [130]. It possesses strong monotherapy efficacy in different preclinical solid tumor and lymphoma models with DDR deficiencies [130,131]. Concerning prostate cancer, potent single-agent anti-tumor activity was demonstrated in vivo. Furthermore, additive to synergistic effects were observed in preclinical in vitro and in vivo models when combining BAY 1895344 with the AR antagonist darolutamide [131]. In addition, a triple combination of BAY 1895344, darolutamide, and radiation therapy achieved better activity than the respective dual combinations and even castration [132]. Furthermore, synergistic effects of BAY 1895344 in combination with radium-223 were reported in an in vivo prostate cancer model mimicking bone metastases [133]. Several phase 1 clinical trials have been initiated to evaluate BAY 1895344 in patients with advanced tumors either in monotherapy or in combination with the PD-1 immune checkpoint inhibitor pembrolizumab or with the PARP-1 inhibitor niraparib (Table 2). In the monotherapy first-in-human trial, BAY 1895344 was well tolerated at biologically active doses and showed promising anti-tumor activity in heavily pre-treated patients with different histologies and DDR defects, including ATM aberrations [134].

### 3.4. ATM Inhibitors

ATM has a central function in DSB repair and is recruited by the MRN complex. It phosphorylates numerous downstream substrates, including CHK2, MDC1, and the histone H2AX [8,135]. Several ATM inhibitors have been described and first clinical trials were initiated [135] (Table 2). AZD0156 is a potent ATM inhibitor with efficacy in several mouse tumor models, especially in combination with olaparib or irinotecan [136]. Treatment with AZD0156 and olaparib leads to improved efficacy in patient-derived TNBC models [137]. A clinical phase 1 study to evaluate AZD0156 alone or in combination with olaparib or irinotecan in subjects with advanced tumors has just been started. AZD1390 is a potent and selective ATM inhibitor which was optimized to exert high brain penetration [138]. It radiosensitizes glioma and lung cancer cell lines in vitro, especially those with a p53 mutation and induces tumor regression in vivo in an orthotopic lung cancer model with brain metastases [138]. AZD1390 combined with radiation therapy is currently being evaluated in glioblastoma patients. Another ATM inhibitor, M3541, sensitizes tumor cells to radiation and topoisomerase inhibition, showing synergistic anti-tumor effects with radiotherapy in tumor xenograft models [139]. It is currently under investigation in combination with radiotherapy in a dose–escalation study in subjects with solid tumors. Generally, only few data are published concerning the impact of ATM inhibitors on prostate cancer. ATM deficiency due to complete protein loss or mutation has been observed in approximately 5–10% of advanced mCRPC tumors [140]. Defective ATM activity alters the DDR and sensitizes to ATR inhibition as demonstrated in preclinical prostate cancer models [141]. The ATM inhibitor KU-60019 significantly reduces growth of a PTEN-deficient prostate cancer xenograft [142]. Also, a combination of KU-60019 with an AR antagonist results in cell death in androgen-sensitive as well as CRPC cell lines and synergistically inhibits growth of the 22Rv1 tumor xenograft model which does not respond to the respective single-agent treatments [143,144].

### 3.5. CHK1 Inhibitors

CHK1 and CHK2 are the respective downstream targets of the two major signaling cascades driving the DDR, namely, the ATR and ATM kinase pathways. Cross-regulation in the case of deficiency of either ATM or ATR has also been reported [145]. The first CHK inhibitors described were unspecific and elicited significant side effects in the clinic [10]. Selective CHK1 inhibitors that are currently in clinical evaluation include prexasertib (LY2606368), GDC-575, and CCT245737. Prexasertib is the most advanced compound and is being tested in several phase 2 studies including trials focusing on TNBC and on mCRPC (Table 3) [146]. The compound reduces HR efficiency and shows synergistic antiproliferative activity in combination with olaparib in TNBC cell lines [147]. First clinical data with prexasertib showed only limited monotherapy efficacy in TNBC patients with wild-type BRCA, suggesting that combinations with other drugs will be needed for improved therapeutic activity [148]. LY2880070 was evaluated in a phase 1b study in combination with gemcitabine for treatment of patients with advanced cancers, including breast cancer, to determine the optimal dosing schedule, and a partial response in an ovarian cancer patient was reported [149]. MU380 strongly blocks the growth of docetaxel-resistant prostate cancer xenografts, either as a single agent or when combined with gemcitabine [150].

### 3.6. WEE1 Inhibitors

Activation of the G2/M cell cycle checkpoint to allow time for the DDR process is controlled by the WEE1 kinase. WEE1 inhibitors are expected to be efficacious in G1 checkpoint-deficient tumors such as those carrying p53 mutations [151]. Adavosertib (AZD1775) is the most advanced WEE1 inhibitor and has been evaluated in preclinical studies in diverse tumor types [152]. It shows strong in vitro and in vivo efficacy in TNBC models, especially in combination treatment with capecitabine [153]. Further, the combination of AZD1775 with a single high-dose gamma irradiation delays growth of breast cancer models in vivo and reduces the radiation-induced PD-L1 expression [154]. Combination treatment with adavosertib and the BCL2 inhibitor navitoclax leads to inhibition of tumor growth in a small-cell neuroendocrine patient-derived prostate cancer xenograft model [155]. Adavosertib is currently in clinical development for different tumor indications, including breast cancer. Initial data from a phase 2 study assessing efficacy in tumors with p53 mutations in ovarian and small cell lung cancer patients, however, showed only a limited benefit (Table 3) [156,157].

### 3.7. CDK12 Inhibitors

CDK12, acting in a complex with cyclin K, phosphorylates the C-terminal domain of RNA polymerase II and plays an essential role in controlling the transcription of numerous central DDR genes, including BRCA1 and ATR, as well as in the regulation of cellular CHK1 protein levels [158,159,160]. CDK12 also has a central function in controlling the expression of centrosome, centromere, and kinetochore proteins, underpinning its importance in the maintenance of genomic stability [159]. Indeed, CDK12 inactivation is observed in different cancer types, including mCRPC and breast cancer [45,161,162,163]. Conversely, amplification and oncogenic function of CDK12 have been reported for a HER2-positive breast cancer model [164]. Prostate cancer patients with mutated CDK12 have a more severe disease progression [165]. It is hypothesized that patients with CDK12-deficient tumors may benefit from immune checkpoint inhibitor treatment due to the increase in tumor immunogenicity [166,167]. Loss of CDK12 also increases the anti-tumor activity of combination treatments with PARP-1 and CHK1 inhibitors in TNBC models [168,169]. THZ531 is a selective inhibitor of CDK12 and the related kinase CDK13 which covalently binds to a cysteine residue outside of the ATP-binding pocket to inhibit enzymatic activity. This ultimately hinders the expression of DDR and transcription factor genes, resulting in blockade of tumor cell proliferation as demonstrated in leukemia cells [170]. SR-4835 is another selective but competitive CDK12 inhibitor which is able to downregulate the expression of DDR genes [56]. Synergistic anti-proliferative effects are seen for SR-4835 when combined with cisplatin, irinotecan, doxorubicin or olaparib, as demonstrated in TNBC models in vitro. Anti-tumor efficacy is furthermore observed in vivo following SR-4835 plus cisplatin or irinotecan treatment of TNBC patient-derived models. Future clinical studies with selective inhibitors will show the validity of this approach for cancer treatment.

## 4. Blocking DNA Repair in Prostate and Breast Cancer to Improve Chemotherapy, General Radiation, and Targeted Radiation Therapy

Cytotoxic chemotherapy and radiation treatment are mainstay treatments of many cancer types. They may cause severe DNA damage directly by induction of breaks or other lesions, or indirectly due to the formation of reactive oxygen species, and this will mostly affect rapidly dividing cancer cells [62,171,172]. For instance, platinum derivatives form intra-strand DNA cross-links that the cells need to amend by NER, single-strand DNA repair or the Fanconi anemia pathway. Alkylating agents bind to one or both DNA strands and cause them to break upon cell division. Antimetabolites lead to stalling of the replication fork which may induce different DNA lesions. Topoisomerase inhibitors cause DNA breaks and ultimately cell death.

Radiation therapy induces different DNA lesions, including base damaging, SSBs, and less frequently DSBs [171,173]. DSBs can be indirect and occur during replication in case the initial damage was not mended. In contrast, targeted alpha therapies like radium-223 predominantly act via the induction of difficult-to-repair, clustered DSBs [174]. Repair of the induced damage can take place via different cellular pathways so that a simultaneous targeting of the key DDR enzymes mentioned above represents a promising approach currently under intensive evaluation [62]. Also, resistance to radiotherapy is linked to enhanced DNA repair capacities so that a combination with DDR inhibitors may significantly ameliorate the outcome [175]. Clearly, improved efficacy of radiotherapy should not be deleterious to neighboring normal tissues and the therapeutic window needs to be carefully assessed.

Concerning prostate cancer, external and internal radiation therapy are commonly used in patients with localized or locally advanced tumors [176,177]. Importantly, androgens cause radioresistance in prostate cancer by upregulating DNA repair genes such as DNA-PKcs [178]. Numerous preclinical studies have evaluated the potential of combining radiation with different targeted agents such as antagonists of the AR [131,179,180] or inhibitors of PARP-1 [181], ATM [182] or ATR [131]. Several clinical studies evaluating the benefit of radiotherapy applied together or after sensitizing agents are currently ongoing [177]. The shift towards targeted radiotherapy for precision rather than systemic DNA damage bears great promise in cancer treatment as it will specifically target the tumor while sparing other tissues, and a number of clinical studies are now ongoing to address this therapeutic option. Beta emitters are characterized by low linear energy transfer and a long range, so that large tumors can potentially be addressed, but adjacent normal tissue like bone marrow might be hit, too [62]. The beta emitter lutetium-177, coupled to PSMA-617, showed encouraging responses in mCRPC patients and is currently under evaluation in a phase 3 pivotal clinical trial [183]. Alpha emitters couple high linear energy transfer with short-range effects and lead to complex DSB formation in close proximity to the radiation source [62]. Radium-223 was the first alpha emitter approved for CRPC patients with bone metastases and no evidence of visceral metastases, based on a significant prolongation of overall survival [184]. After its preferential uptake in osteoblastic bone metastasis, it leads to DNA DSBs and ultimately to cell death of adjacent prostate cancer cells and cells of the tumor microenvironment like disease-promoting osteoblasts [185]. Retrospective studies may indicate that mCRPC patients with germline or somatic mutations in DDR genes have an improved response and a longer overall survival following radium-223 treatment [186,187]. Several clinical trials are currently ongoing in mCRPC patients where radium-223 is combined with different agents including the PARP-1 inhibitors olaparib and niraparib [188]. The alpha emitter actinium-225, coupled to PSMA-617, has been evaluated in mCRPC patients but limiting side-effects were observed [189,190]. For actinium-225, coupled to the anti-PSMA antibody J591, clinical dose–escalation data in progressive mCRPC patients are available and a recommended phase 2 dose was defined [191]. Thorium-227 linked to a PSMA-targeting antibody has shown promising efficacy in preclinical prostate cancer models and a phase 1 trial is now ongoing in mCRPC patients [192].

In the case of breast cancer, several clinical studies combining chemotherapy or radiation therapy with PARP-1 inhibitors are ongoing but limiting adverse events have been reported in several instances [193]. A study in TNBC patients with residual disease shows that sensitization to radiation therapy occurs following treatment with the ATR inhibitor VX-970 [194]. Preclinical data show that the radiosensitivity of TNBC cell lines increased after treatment with the CHK1 inhibitor MK-8776 [195]. Concerning targeted alpha therapy, a number of clinical studies are ongoing in breast cancer [196]. Thorium-227 coupled to trastuzumab showed encouraging preclinical efficacy in breast cancer models [197] but no recent data are available. Actinium-225 conjugated to the IGF-1R monoclonal antibody cituxumab prolongs the survival of mice bearing a TNBC xenograft [198].

Altogether, these results underline the potential of chemotherapy or targeted radiotherapy conjugated with agents targeting DNA repair. The selection of the best combination regimens remains a challenge and biomarker-driven approaches will be essential in this regard.

## 5. Conclusions and Perspectives

The major progress made in recent years in understanding the cellular machinery involved in DNA repair and its role in tumors has led to the discovery of novel cancer targets and thereafter of specific inhibitors. The PARP-1 inhibitors are now approved for ovarian, breast, and prostate cancer harboring BRCA mutations, and more clinical studies are currently ongoing. Compounds targeting other important DDR players, such as DNA-PKcs, ATM, ATR, CHK1, and WEE1, are now also in clinical testing for different cancer types, including prostate and breast cancer, and this will hopefully lead to additional drug approvals soon. Also, numerous studies combining anti-hormonal approaches with DDR-targeting agents are being conducted in steroid hormone-dependent tumors and, here, also novel successful therapeutic approaches should soon be identified. Concerning TNBC, there are currently few treatment options available apart from chemotherapy and radiation, but clinical studies to evaluate PARP inhibitors are currently ongoing [199,200]. In addition, agents targeting kinases involved in the DDR, such as ATR, CHK1, and WEE1, are being tested for activity in preclinical TNBC models, which will hopefully lead to novel therapeutic options soon [199]. As frequently seen for other cancer therapies, toxicity linked to new therapies may be limiting and needs to be evaluated carefully by testing specific dosing schedules to determine the optimal therapeutic window. Combinations with targeted radiation therapy are furthermore being evaluated and also, here, the optimal treatment schedule needs to be precisely defined in order to increase response while minimizing adverse events. Intensive efforts are ongoing to identify biomarkers to ascertain that the target is selectively hit and to predict and monitor the response to these novel agents [18,172,201,202]. Finally, the mechanisms underlying treatment resistance, as already observed for PARP-1 inhibitors, need to be understood and the steady progress made in different “omics” approaches to analyze tumor and blood samples from patients along the treatment time will play an important role [23,203,204,205]. Proper integration of these analyses will help to develop strategies to delay or even overcome resistance and ultimately improve the outcome for prostate and breast cancer patients treated with drugs that address the DDR.

## Figures and Tables

**Table 1:** Selected clinical trials evaluating poly(ADP-ribose) polymerase 1 (PARP-1) inhibitors in prostate or breast cancer.

Compound	Additional Treatment	Condition	Inclusion Criteria	Phase	Identifier
Olaparib	Pembroli-zumab	Prostatic neoplasms		3	NCT03834519
Olaparib	Cediranib	mCRPC		2	NCT02893917
Olaparib	AZD6738	mCRPC		2	NCT03787680
Olaparib	Durvalumab	Castration-sensitive nmPC	DDR mutations	2	NCT03810105
Olaparib	Radium-223	mCRPC	Bone metastases	1/2	NCT03317392
Rucaparib		mCRPC	HR deficiency	3	NCT02975934
Rucaparib	Enzalutamide	mCRPC	Resistance to testosterone deprivation	3	NCT04455750
Rucaparib		mCRPC	HR deficiency Post-docetaxel and carboplatin	2	NCT03442556
Rucaparib		Non-metastatic prostate cancer	BRCAness genotype	2	NCT03533946
Niraparib		mCRPC	DNA repair anomalies	2	NCT02854436
Niraparib	Abiraterone, leuprolide, radiotherapy	High-risk and node-positive prostate cancer		½	NCT04194554
Veliparib	Abiraterone	mCRPC		2	NCT01576172
					
Olaparib		mBC	Germline BRCA positive	3	NCT02000622
Olaparib	Platinum-based neoadjuvant chemotherapy	TNBC	Germline BRCA positive	2/3	NCT03150576
Niraparib		TNBC	HER2 negative Germline BRCA positive	3	NCT01905592
Veliparib	Carboplatin Paclitaxel	mBC	HER2 negative	3	NCT02163694
Talazoparib		mBC	BRCA mutation	3	NCT01945775

Abbreviations: mCRPC, metastasized castration-resistant prostate cancer; nmPC, non-metastasized prostate cancer; mBC, metastasized breast cancer; TNBC, Triple-negative breast cancer; DDR, damage response; HR, homologous recombination; HER2, human epithelial growth receptor 2.

**Table 2:** Selected clinical trials evaluating DNA-dependent protein kinase catalytic subunit (DNA-PKcs), ataxia–telangiectasia-mutated (ATM) or ATM and Rad3-related (ATR) inhibitors in solid tumors including prostate or breast cancer.

Compound	Target	Additional Treatment	Condition	Inclusion Criteria	Phase	Identifier
Nedisertib	DNA-PKcs	Radium-223 Avemulab	mCRPC		1/2	NCT04071236
AZD7648	DNA-PKcs	Doxorubicin Olaparib	Advanced cancers		1/2	NCT03907969
VX-984	DNA-PKcs	Chemotherapy	Advanced solid tumors		1	NCT02644278
CC-115	DNA-PKcs	Enzalutamide	CRPC		1	NCT02833883
Berzosertib	ATR	Radiation therapy	Breast cancer		1	NCT04052555
Berzosertib	ATR	Carboplatin	mCRPC		2	NCT03517969
Ceralasertib	ATR	Olaparib	mCRPC		2	NCT03787680
Ceralasertib	ATR	Olaparib	TNBC		2	NCT03330847
Ceralasertib	ATR	Olaparib	Advanced breast cancer	Germline BRCA mutation	2	NCT04090567
M4344	ATR	Chemotherapy	Advanced solid tumors		1	NCT02278250
BAY 1895344	ATR		Advanced solid tumors and lymphomas		1	NCT03188965
BAY 1895344	ATR	Chemotherapy	Advanced solid tumors		1	NCT04491942
BAY 1895344	ATR	Niraparib	Advanced solid tumors		1	NCT04267939
BAY 1895344	ATR	Pembrolizumab	Advanced solid tumors		1	NCT04095273
AZD0156	ATM	Olaparib Irinotecan Fluorouracil Folinic acid	Advanced cancer		1	NCT02588105
M3541	ATM	Radiotherapy	Solid tumors		1	NCT03225105

Abbreviations: DNA-PKcs, DNA-dependent protein kinase catalytic subunit; ATM, ataxia–telangiectasia-mutated kinase; ATR, ATM and Rad3-related kinase; CRPC, castration-resistant prostate cancer; mCRPC, metastasized CRPC; TNBC: triple-negative breast cancer.

**Table 3:** Selected clinical trials evaluating CHK1 or WEE1 inhibitors in solid tumors including prostate or breast cancer.

Compound	Target	Additional Treatment	Condition	Inclusion Criteria	Phase	Identifier
Prexasertib	CHK1		mCRPC TNBC Ovarian cancer	BRCA mutation	2	NCT02203513
Prexasertib	CHK1	LY3023414	TNBC		1	NCT04032080
LY2880070	CHK1	Gemcitabine	Solid tumors		1	NCT02632448
Adavosertib	WEE1		Prostate cancer		2	NCT03385655
Adavosertib	WEE1	Olaparib	TNBC		2	NCT03330847
AZD1775	WEE1	Cisplatin	TNBC		2	NCT03012477

Abbreviations: mCRPC, metastasized breast cancer; TNBC, triple-negative breast cancer.
